# The Sunshine Act: Commercial conflicts of interest and the limits of transparency

**Published:** 2014-01-14

**Authors:** Mark Wilson

**Affiliations:** Mark Wilson is a bioethicist living in Guelph, Ontario, Canada.

Godlee is referring to the Physician Payment Sunshine Act, which became federal law in the United States in January 2012.^2^ The Act requires drug and medical manufacturers who participate in federal programs to report annually on direct payments and other transfers of value made to physicians and teaching hospitals. As part of the disclosure mandated by the Act, information on amounts received by individual physicians will be made available on a publicly accessible website beginning in September 2014.

The aim of the legislation is to tackle the issue of commercial conflicts of interest (COIs), which has been a source of heated debate in the medical community for well over a decade. Media coverage of commercial conflicts within medicine focused greater public attention on the issue and raised concern about the integrity of medical information.^3^ In 2009, the US Institute of Medicine weighed in with a report that described commercial COIs within medical practice and education as widespread and often hidden from public view.^4^ The report stressed that financial ties can exert undue influence on physicians' judgments, research, and practice, thus jeopardizing patient care, scientific integrity, and objectivity in education. It recommended that medical education should be free of industry sponsorship and that experts on panels who establish clinical guideline practices be prohibited from having commercial COIs. Both recommendations were intended to ensure greater public trust in medicine.

Another recommendation was for the implementation of policies that would make financial ties between industry and the medical community more transparent to the public. This particular recommendation was welcomed by Senator Chuck Grassley, co-author of the Sunshine Act and a champion of the view that metaphorical sunshine—disclosure—is a powerful aid to accountability. Grassley was instrumental in heightening public concern about commercial COIs and the need for greater accountability. He spearheaded investigations that brought financial ties out of the shadows and exposed egregious violations of federal conflict-of-interest reporting requirements by influential physicians in eminent academic institutions.^5^ These investigations reinforced the perception that commercial COIs were ubiquitous and poorly managed, and that they required better oversight to mitigate their effects.

Against this backdrop, the Sunshine Act gained political momentum and eventually became law as part of the Affordable Care Act of 2010. Through its requirements for disclosure of commercial COIs, the Act is seen as a tool to empower patients to decide whether physician prescribing patterns are influenced by financial interests in the pharmaceutical and medical device industries.^6^

There are no signs that any legislative measures along the lines of the Sunshine Act are imminent in Canada, although there have been calls for legislators in Australia,^7^ the United Kingdom,^8^–^9^ and the European Union to follow the United States' lead.^1^ Despite this international interest in the Sunshine Act, psychological, institutional, and behavioural research suggests that this kind of legislation, with its stress on transparency, will not effectively combat commercial COIs. Indeed, there is good reason to believe that the Sunshine Act will only preserve the status quo.

Various researchers have examined the unconscious character of bias and the limits of disclosure as a standalone measure to address commercial COIs.^10^–^12^ Even though the view that disclosure is a necessary but insufficient means of combatting commercial conflicts has gained a toehold in the medical literature,^13^–^14^ it has never gained traction in public policy. The issue over mitigating the influence of commercial interests was framed primarily around disclosure:^15^ whether to disclose a commercial COI; what kind and how much information should be disclosed; whether disclosure should be mandatory; and whether disclosure of a commercial COI was too intrusive.^16^–^20^ Public attention became fixed on ensuring commercial COIs did not remain hidden. Meanwhile, scant attention was paid to the unconscious nature of bias, the potential for bias to become institutionally embedded, and its ability to compromise the integrity of medical data. Ironically, the stress on disclosure hid the real problem: the existence of commercial conflicts and their consequences.^15^,^21^ In short, a preoccupation with disclosure hijacked the debate and sidetracked the public interest. Jerome Kassirer, former chief editor of the *New England Journal of Medicine*, critiqued this fixation on "the wrong problem"^15^ and expressed concern that the need to eliminate commercial conflicts, especially from oversight bodies that assess the integrity of medical data, was being excluded as a public policy option.^21^

This concern was shared by analysts in other sectors who examined the unconscious nature of bias and the ineffectiveness of disclosing commercial COIs.^22^–^23^ Decades of market scandals reveal a history of compromised auditor independence arising from consultancies with corporate clients.^24^ Wall Street's attempt to manage the problem by disclosing these conflicts failed to prevent further scandals involving tainted accounting audits. Calls to eliminate commercial conflicts altogether^25^–^26^ fell on deaf political ears, partly because of lobbying by powerful interest groups.^24^,^27^ Then emerged a fear that more "predictable surprises" would ensue—that is, situations in which the gravity of avoidable crises is underestimated in order to satisfy economic and social policies.^28^ On Wall Street this fear was borne out by the 2008 global financial crisis, in which auditors with consulting relations with major banks signed off on overstated bank balance sheets and fictitious earning statements. Calls to end these commercial conflicts and for genuine auditor independence continue.^29^–^30^

The relevance of the Wall Street experience was not lost on members of the medical, business, and bioethics academic communities who examined the unconscious nature of bias in the context of commercial COI and institutional corruption; for instance, a public policy symposium held at Harvard University examined the cognitive mechanisms of bias and the sometimes paradoxical effects of disclosure.^31^ However, such subleties were ignored by the framers of the Sunshine Act, despite the fact that disclosure has been demonstrated to be a flawed accountability tool on Wall Street.

What the Sunshine Act achieves is to shift the burden of managing the problem of commercial conflicts onto the shoulders of a powerless consumer public.^39^ With respect to health care—and the science that underpins it—the public lacks the specialized training, practice, and knowledge required to effectively assess the integrity of medical data and the various contexts in which it is embedded. And simply disclosing a commercial COI to the public does not provide the necessary skills to assess its implications. There is a good reason why medical expertise is required to assess the integrity of medical information. The problem is that independence and objectivity can, and have been, compromised by commercial COIs.^33^–^35^ In short, the Sunshine Act fails to fix the problem;^36^ instead, it mythologizes transparency.

This is not surprising, given the dominant political approach to the problem. Systemic commercial COIs continue to flourish on Wall Street, despite a near market collapse.^37^ With this in mind, it is difficult to gauge what kind of tipping point will rally politicians to help turn the tide in medicine. Furthermore, it is not by chance that the Sunshine Act mirrors the way US politicians tackle the issue of financial campaign contributions from special interest groups. Public disclosure of campaign contributions plays a key role in managing the issue.^38^ The Sunshine Act is symptomatic of a self-serving political culture that lacks the will to end commercial conflicts.

Until politicians end their own commercial COIs, the Sunshine Act will likely remain the governance order of the day. The public will have to shoulder the regulatory weight and negative consequences of an unresolved problem, just as it bailed out the banks during the financial crisis while lacking the power to curb commercial conflicts on Wall Street. As a political response to a systemic problem, the Sunshine Act echoes the marketing mantra that "consumers know best" and that the market is the best information medium and solution to public policy issues.^39^ The result is a false sense of public empowerment and a pseudo accountability that sustains the status quo.

Greater transparency in medicine should be welcomed. A world in which the best experts in science and health care are free of commercial influence is surely desirable. But the Sunshine Act fails to disinfect a medical and political landscape awash in commercial conflicts. Consumers beware: the predictable surprises are becoming ever more common.
